# A Comprehensive In Silico Analysis of the Functional and Structural Impact of Nonsynonymous SNPs in the ABCA1 Transporter Gene

**DOI:** 10.1155/2014/639751

**Published:** 2014-08-19

**Authors:** Francisco R. Marín-Martín, Cristina Soler-Rivas, Roberto Martín-Hernández, Arantxa Rodriguez-Casado

**Affiliations:** ^1^Department of Production and Characterization of New Foods, Institute of Food Science Research (CIAL), UAM-CSIC, Campus de Cantoblanco, 28049 Madrid, Spain; ^2^IMDEA Food Institute, Campus de Cantoblanco, 28049 Madrid, Spain; ^3^Nutritional Genomics of the Cardiovascular Disease and Obesity, IMDEA Food Institute, Carretera Cantoblanco 8, 28049 Madrid, Spain

## Abstract

Disease phenotypes and defects in function can be traced to nonsynonymous single nucleotide polymorphisms (nsSNPs), which are important indicators of action sites and effective potential therapeutic approaches. Identification of deleterious nsSNPs is crucial to characterize the genetic basis of diseases, assess individual susceptibility to disease, determinate molecular and therapeutic targets, and predict clinical phenotypes. In this study using PolyPhen2 and MutPred in silico algorithms, we analyzed the genetic variations that can alter the expression and function of the ABCA1 gene that causes the allelic disorders familial hypoalphalipoproteinemia and Tangier disease. Predictions were validated with published results from *in vitro, in vivo*, and human studies. Out of a total of 233 nsSNPs, 80 (34.33%) were found deleterious by both methods. Among these 80 deleterious nsSNPs found, 29 (12.44%) rare variants resulted highly deleterious with a probability >0.8. We have observed that mostly variants with verified functional effect in experimental studies are correctly predicted as damage variants by MutPred and PolyPhen2 tools. Still, the controversial results of experimental approaches correspond to nsSNPs predicted as neutral by both methods, or contradictory predictions are obtained for them. A total of seventeen nsSNPs were predicted as deleterious by PolyPhen2, which resulted neutral by MutPred. Otherwise, forty two nsSNPs were predicted as deleterious by MutPred, which resulted neutral by PolyPhen2.

## 1. Introduction

Nonsynonymous single nucleotide polymorphisms (nsSNPs) are single base changes in coding regions that cause an amino acid substitution in the correspondent proteins. These missense variants constitute the most identifiable group of SNPs represented by a small (<1%) proportion [1]. The nsSNPs might alter structure, stability, and function of proteins and produce the least conservative substitutions with drastic phenotypic consequences [2–5]. Studies suggest that about 60% of Mendelian diseases are caused by amino acid exchanges [6]. Thousands of associations between Mendelian and complex diseases reveal a phenotypic code that links each complex disorder to a unique set of Mendelian loci [7]. Discriminating disease-associated from neutral variants would help to understand the genotype/phenotype relation and to develop diagnosis and treatment strategies for diseases. Nonetheless, the most important application is the evaluation of functional effect and impact of genomic variation, relating interactions with phenotypes translating the finding into medical practices.

ATP-binding cassette transporter ABCA1 gene also known as the cholesterol efflux regulatory protein (CERP) encodes a 220 kDa protein [8]. This protein is crucial for reverse cholesterol transport and is considered as an important target in antiatherosclerosis treatment. ABCA1 mediates the efflux of cholesterol and phospholipids to lipid-poor apolipoproteins (apoA1 and apoE), which form nascent high-density lipoproteins (HDL). ABCA1 resides on the cell membrane and has an extensive intracellular pathway, with rapid movement of the transporter between the cell membrane and intracellular vesicles [9]. ABCA1 is present in higher quantities in tissues that transfer or are involved in the turnover of lipids such as the liver, the small intestine, and adipose tissue [10–12]. As well, lipid export activity of ABCA1 improves the function of pancreatic cells and ameliorates insulin release [13], reduces biliary cholesterol content protecting against gallstone [14], and plays a key role in lipid homeostasis in the lung [15]. Besides, evidence suggest a causal link between ABCA1 as cholesterol transporter and its antitumor activity [16, 17], as well as its implication in brain cholesterol homeostasis [15–20] founding lipid and myelin abnormalities in schizophrenia and Alzheimer's disease [18–20].

**Figure 1:**
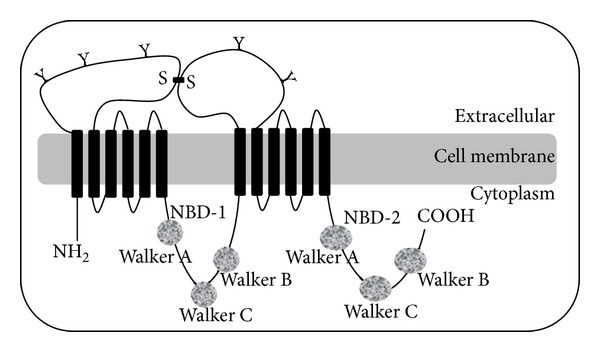
Predicted structure of ABCA1 [21, 23, 105]. The protein consists of 2261 amino acids and comprises 2 halves of similar structure. Each half encodes a transmembrane domain containing 6 helices (1–6 and 7–12) and 1 nucleotide binding domain (NBD-1 and NBD-2), where ATP is bound and utilized as energy for substrate transport across the membrane. Each NBD contains 2 highly conserved peptide motifs known as Walker A and Walker B, which are present in many proteins that use ATP and a Walker C signature unique to ABC transporters. ABCA1 is predicted to have an N terminus oriented into the cytosol and two large extracellular loops that are highly glycosylated and linked by cysteine bonds (Y indicates the glycosylation sites, and S–S indicates predicted disulfide bonds).

Although the entire ABCA1 protein 3D-structure remains unknown electron microscopic studies suggest a structural model consisting of a transmembrane domain (TMD) and a nucleotide-binding domain (NBD) (Figure 1) [14, 21, 22], where an NBD-TMD dimer is the minimum unit required for transport function [22, 23]. Also, X-ray structures are available for different domains in the C-terminus protein essential for lipid efflux activity [24, 25]. Many variants disrupting the normal ABCA1 protein function result in modest or no circulating HDL [26–32]. Cholesterol accumulated within cells produces a toxicity that impairs cell function leading to a diversity of phenotypes, from severe disease states to mild impacts on health. In fact, the ABCA1 variability is associated with myocardial infarction, cancer, type 2 diabetes, and metabolic syndrome [33]. Heterozygous states, nearly one-third of them, are associated with hypoalphalipoproteinemia, known as familial HDL deficiency syndrome (FHA). Two copies cause a more severe syndrome Tangier disease (TD) [34–38] described by reduced HDL-c plasma level (<5%), impaired cholesterol efflux, and a trend to accumulate intracellular cholesterol [34–43]. Indeed, loss of function of ABCA1 mutations in TD patients has a major impact on lipoprotein metabolism. A failure to acquire apolipoproteins leads to a rapid catabolism of lipid-poor apoA1 and accumulation of lipids in macrophages, intestinal cells, platelet, and hepatocytes [34–38, 44]. Compared with unaffected family members, heterozygotes and homozygotes have a more prevalent, premature, and severe atherosclerosis [42].

Because high levels of HDL-c are atheroprotective there is considerable interest in developing agents that act to increase ABCA1 expression and thereby raise plasma HDL-c levels. The nsSNPs are important indicators of action sites and effective potential therapeutic approaches. Therefore, it is crucial to identify deleterious nsSNPs to characterize the genetic basis of diseases, assess individual susceptibility to these diseases, determinate molecular and therapeutic targets, and predict clinical phenotypes. Beyond the genetic level, a disease depends on the sequence and the structural location of the nsSNPs of the protein. While the nsSNPs occur all through the ABCA1 gene, they tend to cluster in the extracellular loops, the NBD, and the COOH-terminal region (Figure 1). In fact, three structural motifs have been functionally associated with disease: the ARA motif, an interface between NBD and TMD that forms a partially buried *α*-helix able to interact with the transmembrane helices, the conserved-loop 1, a allosteric loop between the membrane and globular domains, and the conserved-loop 2, an interaction surface for intracellular partners, critical in ATP-binding.

Even though many of nsSNP (rare or common) found in human ABCA1 have been identified, mainly in the HapMap project (http://hapmap.ncbi.nlm.nih.gov/), the molecular bases relating these variants and the caused phenotypes have not been studied in detail. To explore the effect of the large number of nsSNPs ABCA1 by experimental approaches would be extremely time-consuming and with low statistical chance. Alternatively, bioinformatic approaches, based on the biophysical severity of the amino acid exchange and the protein sequence and structural information, can offer a more feasible phenotype prediction. As such, MutPred (mutation prediction) [45] and PolyPhen2 (polymorphism phenotyping 2) algorithms [46], were used in this study to investigate the impact of all known nsSNPs on ABCA1 protein function. Besides, based on the results of* in vitro, in vivo*, and human studies of this gene in the literature we validated the predictions made by reviewing the effect of the most critical nsSNPs in ABCA1 gene and its pathological consequences.

## 2. Methods

**Figure 2:**
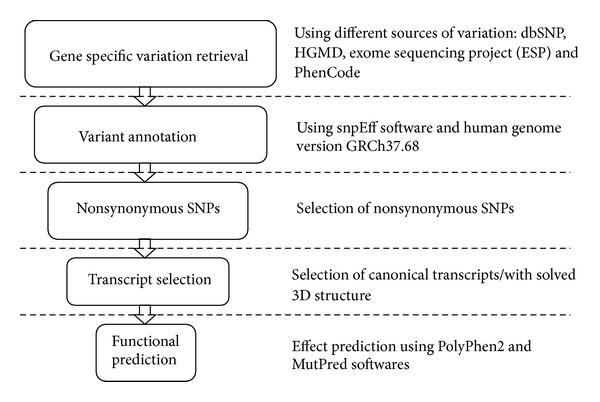
Workflow for predicting the effect of nsSNPs on ABCA1 protein. Gene specific variations were retrieved from the main variation databases. Mutations were annotated and nsSNPs belonging to the canonical transcript were selected in order to run the algorithms for functional prediction.

**Figure 3:**
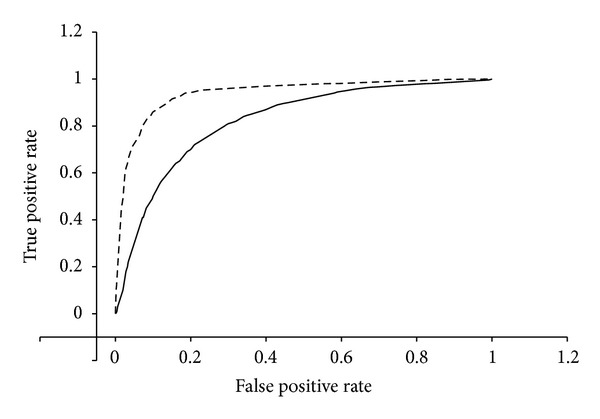
ROC (receiver operating characteristics) curve comparing the performance of MutPred and PolyPhen2 methods in predicting the outcome of nsSNPs. The dashed curve corresponds to MutPred predictions. As the area under the curve (AUC) for the MutPred method predictions is larger than the areas over the curve (AOC) which corresponds to the PolyPhen2 predictions curve, we can confirm that MutPred outperforms PolyPhen2 in predicting the outcome of nsSNPs. The dataset used for the performance comparison was obtained from Varibench as stated in Methods section.

Figure 2 shows the workflow designed to predict the nsSNPs effect on ABCA1 protein. ABCA1 human gene variants including SNPs, short insertions, and deletions were retrieved from Ensemble Variation 72 database 3141. Mutations were annotated using the SnpEff v3.2 toolbox [47] based on the human genome assembly GRCh37.68. Only variants found on the canonical transcript were considered for functional effect prediction, for what we used two different algorithms. PolyPhen2 (http://genetics.bwh.harvard.edu/pph2/) algorithm uses a naive Bayesian classifier to predict allele function based on a combination of sequence and structure-based attributes (if available) [46]. It calculates the probability for a given mutation to be benign, possibly damaging, or probably damaging. Then, we used MutPred (http://mutpred.mutdb.org/) [45] based upon SIFT algorithm [48] and a gain/loss of 14 predicted structural and functional properties. The predicted mutation outcome is based on a random forest (RF) classifier. The MutPred output includes the top 5 property scores and a general score (RF) equal to the probability of amino acid exchange is either deleterious or disease-associated. The ROC curves for both methods were generated using R programming language and the ROCR package (Figure 3) by a variation dataset obtained from VariBench (http://structure.bmc.lu.se/VariBench/) that contains mutations affecting protein tolerance including a neutral set of mutations comprising 17393 human coding nsSNPs and a pathogenic set of 14610 missense mutations obtained by manual curation from the PhenCode database.

Prediction accuracy accomplished by MutPred and PolyPhen2 depends on their specific criterion. Twelve structural and six sequence-based properties were used in this study (Table 1). About 28% of validated nsSNPs in the Human Genome Variation Database are predicted to affect protein function [49]. Similarly, about 25% of nsSNPs affecting protein activity was predicted by PolyPhen2 [49]. MutPred offers classification accuracy with respect to human disease mutations. Considering conservative thresholds on the predicted disruption of molecular function, MutPred generates accurate and reliable hypotheses on the molecular basis of disease for about 11% of known inherited disease-causing mutations [45].

Our MutPred and PolyPhen2 predictions were validated by comparing them with previously obtained results from* in vitro, in vivo*, and human studies of ABCA1 gene in the databases and literature. When a given nsSNP found experimentally to be associated with a remarkable change of phenotype such as altered transporting activity or a disease was predicted by in silico methods as deleterious, it was considered that the prediction on this nsSNPs was correct. The prediction was defined as an error if such a deleterious nsSNP was predicted as tolerant.

## 3. Results and Discussion

The importance of ABCA1 in cholesterol efflux was demonstrated by the identification of ABCA1 mutations in TD and FHA families [34–38]. This has produced extensive research into the possibility to provide protection from atherosclerosis by increasing ABCA1 expression and thereby to raise plasma HDL-c levels. The identification of the large number of alleles for this transporter gene as target directly involved in HDL-c regulation constitutes a significant therapeutic strategy in reducing the risk for atherosclerosis.

### 3.1. Accuracy of the Prediction of the Functional Impact of nsSNPs

**Figure 4:**
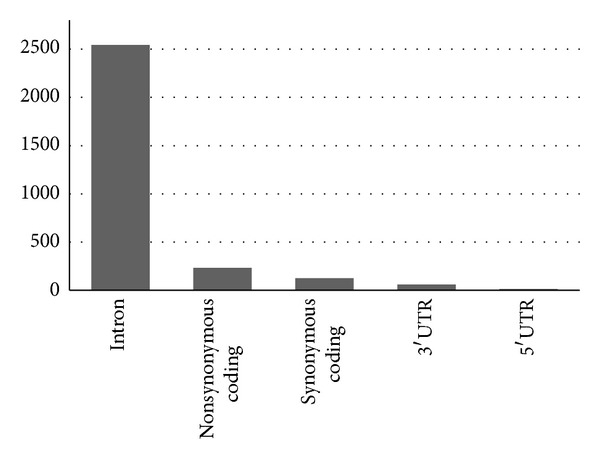
Distribution of ABCA1 nonsynonymous SNPs (nsSNPs), synonymous SNPs (sSNPs), 3′-UTR and 5′-UTR, and intronic SNPs.

**Figure 5:**
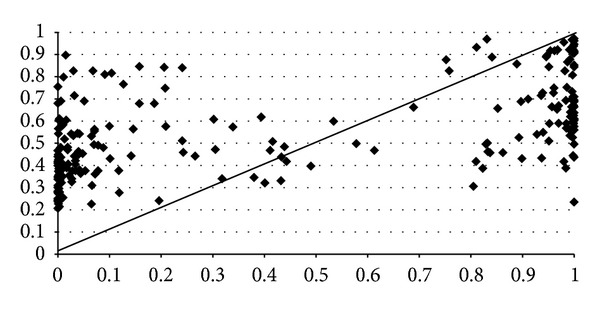
Scatter plot shows the correlation between the predictions made by PolyPhen2 (*x*-axis) and MutPred (*y*-axis) for 233 amino acid substitutions on ABCA1. The diagonal line represents a perfect correlation (=1) between both prediction methods for every mutation. The overall correlation of the predictions made by both methods is high (0.57). The majority of mutations classified as pathogenic by PolyPhen2 with the highest score (=1) are also classified as pathogenic by MutPred but within a score range between 0.51 and 1.

Out of a total of 3141 SNPs in ABCA1 gene retrieved from dbSNP, we found 233 nsSNPs, 126 sSNPs, 59 mRNA 3′-UTR SNPs, 12 mRNA 5′-UTR SNPs, and 2543 intronic SNPs (Figure 4). Among the 233 nsSNPs, MutPred (RF score > 0.5) predicted 122 (52.36%) as deleterious whereas PolyPhen2 (pph2_prob > 0.5) identified 97 (41.63%) as potentially damaging and damaging. Then, once that MutPred was used to predict the nsSNP disease-association probability, the damaging probability of nsSNPs was validated by PolyPhen2. A total of 80 (34.33%) nsSNPs were found to be deleterious by both methods. Among these 80 deleterious nsSNPs, the 29 (12.44%) targeted (MAF/NA) that resulted with high pathological phenotype (probability > 0.8) are C1477R, W590L, W590S, A1046D, N1611D, M1091T, F2009S, N935H, R2081W, R1068H, N935S, R1068C, D1099Y, D1099N, W1699C, W840R, A937V, I1517R, C1660R, R1680W, P1065S, R1615P, T929I, Y2206D, L1379F, T940M, G1216V, Y2178H, and R1680Q. As shown in Table 2, a good correlation index was obtained between the scores observed from the evolutionary-based approach MutPred and the structural-based approach PolyPhen2. As shown in Figure 5, the overall correlation of the predictions made by both methods is high (~0.57). The majority of mutations classified as pathogenic by PolyPhen2 with the highest score (=1) are also classified as pathogenic by MutPred but within a score range between 0.51 and 1. The prediction accuracy depends not only by limitations of the in silico algorithms such as false positive error and interference of redundant motifs but also by the phenotype data from experimental studies [3].

Equally important is to consider the incorrect predictions in order to know the limitations of both algorithms and to suggest how they might be improved. Where MutPred predicts P2150L variant as deleterious, PolyPhen2 indicates a benign amino acid exchange. Conversely, MutPred predicted P85L to be probably damaging, while PolyPhen2 indicates it as neutral. Conflicting results were observed for a few other nsSNPs included in Table 2. A total of seventeen deleterious nsSNPs predicted by PolyPhen2 resulted neutral by MutPred. In contrast, forty two deleterious nsSNPs by MutPred result neutral by PolyPhen2. We have observed (Figure 5, Table 2) that some mutational characteristics of nsSNPs such as C1477F, R666Q, P1475S, G616V, Q2210H, V1806M, and V304M show high PolyPhen2 values but very low MutPred scores due, at least in part, to loss or gain of catalytic residues and disorder and gain of ubiquitination and phosphorylation to the protein. On the other hand, some mutational characteristics of nsSNPs included T459P, A2028V, T774P, Q1279K, N1185K, D917N, E1005K, C887F, D1289N, Q188K, D462G, M1012I, R965C, S1181F, A255T, D457E, R496W, R1341T, R1925Q, R230C, L184S, R999L, and K1974R with low PolyPhen2 values but high MutPred scores produce, however loss of solvent accessibility and of disorder, gain of phosphorylation, and both loss and gain of molecular recognition features (MoRFs) binding, loss and gain of methylation, and loss and gain of helix structure. Both, loss and gain of catalytic residues are actively involved in human inherited disease. Also, the small ubiquitin—a 76 residue *β*-grasp protein—is about 95% conserved from yeast to human. Overall, both gain and loss of a phosphorylation site in a target protein may be important features for predicting cancer-causing mutations and may represent a molecular cause of disease for a number of inherited and somatic mutations. Changes in secondary structure impair large functional alterations, as well as the solvent accessibility degree. Therefore, inaccurate predictions occurred at these sites could be explicated not only for the limited effects of genetic variant but also for gene-environment interactions. Since the MutPred is based on a predicted structure of the protein under study rather than a solved structure as PolyPhen2 and considering the fact that nowadays the ABCA1 protein structure is only partially solved, it makes sense to prioritize the MutPred predictions. This fact was confirmed after evaluating the performance of both methods using a curated nsSNPs dataset with known outcome as a benchmark as shown in Figure 3. We have also observed that mostly, nsSNPs with verified functional effect in experimental studies are correctly predicted as damage variants by MutPred and PolyPhen2 tools. Still, controversial experimental data are obtained for those nsSNPs predicted as neutral by both of these methods.

### 3.2. Functional Assessment of ABCA1 Variants

Disease-causing variants are under strong selective constraints, which determines if mutation frequency will increase, decrease, or change randomly during evolution. Most alterations are deleterious and so are finally removed during purifying selection. Benign mutations can sweep through the population and become fixed contributing to species differentiation. The ABCA1 gene is highly conserved between species. Human ABCA1 is 95.2% identical to mouse, 85.3% to chicken, 25.5% to drosophila, 21.6% to* C. elegans*, and 10.2% identical to fugu. In humans, there is an abundance of common nsSNPs that disrupt sites highly conserved across species and likely to be deleterious [50]. The information of nsSNPs can be used to outline the migration patterns of ancient humans and the ancestry of modern humans. Causal nsSNPs in single gene disorders are sufficient to impart large effects. Instead, complex traits are due to a much more complicated system of causative mechanisms that in aggregate increase the probability of disease. Genome-wide association studies reveal common genetic variants effects (common disease/common variant hypothesis) in complex traits. However, where common nsSNPs account for a relatively slight heritability of the traits, rare variants might produce large effects on the phenotype (rare variant/common disease hypothesis). The frequency range includes alleles that are exceptionally rare and even unique to an individual genome to be extremely common. Most deleterious nsSNPs are retained at low-population frequencies due to negative selection. Thus, variants with large effect tend to be rare and those that exert weak effects are more common. It is worthy to note that rare alleles can also have weak effect or no effect. A specific locus may contain numerous rare alleles, so there may be many rare variants with large effect and a few common variants with weak effects. Although it has not yet been possible to determine whether other variables are associated with specific nsSNPs frequencies, variants within metabolic genes are not randomly distributed along the human population but follow diverse ethnic and/or geographic-specific patterns. It has been reported [51] that a significant proportion (~16%) of individuals with low HDL-c from the general population has the rare sequence of 25 variants in ABCA1 gene (Table 2, MAF ≤ 0.01). However, consistent with MutPred and PolyPhen2 only nine of them, N1800H, W590L, S1731C, C1477R, D1706N, R1615P, R638Q, T2073A, and A1670T, are predicted as functionally impaired. Some deleterious mutations from some other genes have reached intermediate to high frequencies. Specifically, the ancestral APOE4 allele, remains higher in populations like Pygmies (0.41), Khoi San (0.37), Papuans (0.37), some Native Americans (0.28), Lapps (0.31) and aborigines of Malaysia (0.24), and Australia (0.26) [52]. The exposure of APOE4 to the current environmental conditions could have rendered it a susceptibility allele for cardiovascular and Alzheimer diseases. However, the prediction for variant within ABCA1 gene indicates lack of harmful alleles to MAF ≥ 0.01. Therefore we have evaluated and contrasted the predictions made for nsSNPs (rare/common) most widely studied for their role in cholesterol pathway by reviewing the effect of the most significant nsSNPs in ABCA1 gene and its pathological consequences.

#### 3.2.1. Accurate Prediction of the Functionally Deleterious nsSNPs in the ABCA1 Gene

The** N1800H** ABCA1 has been fully characterized showing a complete lack of protein function in terms of cholesterol efflux and HDL production [53, 54]. Unlike the WT (wild-type), which is found at the endoplasmic reticulum and plasma membrane, N1800H is accumulated intracellularly [54]. Even similar physicochemical properties (polar, medium size) of exchanged residues the N1800H nsSNP, located between transmembrane domains [54], is a critical site for protein function. Scores from in silico methods predict the N1800H variant as highly deleterious.

The** W590L** was never studied, but the** W590S** ABCA1 variant affecting the same position is extensively known [54]. Distribution of W590S is identical to WT [55] as well as apoA1 binding activity [54–57]; however it shows defective lipid transport [54, 56, 58, 59]. Since multiple alignments often show a leucine residue in this position, it could be assumed that W590L had a similar behavior or even a lower impact than W590S on the protein function. Both W590S and W590L were predicted as deleterious nsSNPs with loss of functionality.

Studies indicate that** S1731C** variant alters the activity of ABCA1 protein [27, 51, 60]. This allele is present in French-Canadian families with low HDL-c levels [27] but not in subjects with normal [60] or high [51] HDL-c levels. Compared with WT, heterozygous show decreased ~60% the cholesterol efflux activity [27, 51, 61]. Interestingly, some but not all families harboring S1731C also carried the 2144X stop mutation [60] able to produce the most severe effects on HDL-c levels and on cholesterol efflux [62]. These data along with our in silico predictions indicate that conserved S1731C is highly likely to affect protein function.

**S1506L**,** Q597R**, and** C1477R** variants are linked to TD and FHA and found in tumor cancer [54]. Normal function of ABCA1 inhibits tumor growth in human cancer cells [54]. However, although expressed to similar levels as WT, these alleles show deficient cellular cholesterol efflux and HDL production and do not decrease tumor growth [17]. The three are located intracellularly but C1477R is also found in membrane [54], which indicates that membrane localization is essential but not sufficient for apoA1 binding [54, 63]. In fact, ApoA1 binds to ABCA1 protein oligomers but not with monomers [64]. Thus, conformation changes in binding sites might be produced by these nsSNPs found as deleterious by our in silico analysis.

The** R587W** reaches the cell surface but reduces the apoA1 binding efficiency ~50% [56]. Others studies indicate that this allele is mainly retained intracellularly decreasing cholesterol efflux and apoA1 binding ~75% [54]. Severe HDL deficiency [34] and premature CVD is caused by R587W [65]. This variant is highly conserved during evolution, and the in silico analysis predicts it as strongly damaging and disease-associated. Besides to be related with TD, the R587W as well as W590S variants are linked to AD. As the WT, these mutants significantly reduce A*β*-peptide synthesis ~45% [66], but increase by ~2-fold (R587W) and by 25% (W590S) amyloid precursor protein intracellular domain, a major cytotoxic of AD [66].

The** A1046D**, localized between conserved motifs [54, 67], shows an intermediate phenotype caused by its limited presence in the plasma membrane. This variant shows reduced apoA1 binding efficacy, poor HDL-c, and folding protein alteration. Both in silico methods predict A1046D as a functional residue with a probability to be deleterious very close to 1.

According to literature,** N1611D** is associated to probable atherosclerosis [62, 68]. The mutated protein expression was comparable to WT although cholesterol efflux from the cells was markedly reduced. Our theoretical results indicate very high probabilities of this nsSNP being deleterious, which indicate an adverse and potential harmful effect on ABCA1 function.

The** M1091T** variant exerts a dominant-negative impact on ABCA1 function with severe phenotype observed in subjects carrying this variant [42, 54, 62]. It is retained intracellularly preventing the protein from reaching the membrane [54]. In heterozygous, M1091T is lowered by 50% HDL and inhibits apoA1 binding and cholesterol efflux [54]. From evolutionary path, the inherited residue at this position has been methionine. Among related homologues ABCA2 and ABCA4 share a methionine at this position, while ABCA7 substitutes a leucine. Consistent with this fact, ABCA1 and ABCA7 are functionally divergent, with ABCA7 easing the efflux of phospholipids but not cholesterol [69]. Despite the modest conservation at this position, located in a critical cluster at the C-terminal region, in silico data suggest a severe-negative impact.

The** F2009S** is conserved between human and mouse, that along with the exchange from large size and aromatic (F) to small size and polar (S) explicates its reduced cholesterol efflux, low HDL-c, and apoA1 levels [70]. The functional effect produced by F2009S variant is consistent with our prediction made by PolyPhen2 and MutPred indicating a deleterious mutant.

The** N935S** variant is found intracellularly [54] in subjects without risk of premature atherosclerosis but with extremely low levels of HDL and signs of severe dementia and amyloid depositions in the brain [71, 72]. This variant was predicted as deleterious by the used methods.

**R1068H** mutation is located within the first ATP-binding domain. It is identified in TD homozygous [73]. Since the R1068H mutation is likely to produce a dysfunctional protein, one would expect it to be associated with FHA in the heterozygous state [73]. Residue R1068 is located in an *α*-helix of the Walker B motif in the NBD, vulnerable to interaction with D1092 and E1093 [74]. Homology modeling of the ABCA1 protein showed that the R1068H mutation disrupts the conformation of NBD. Functional studies of R1068H showed a lack of cholesterol efflux activity due to defective transference to the plasma membrane, mainly caused by impaired oligomerization [74]. The in silico analysis predicts a high possibility for R1068H to be damaging. Besides, a different mutation of this position,** R1068C**, predicted as a deleterious by our methods, has been reported in a compound heterozygote with almost no HDL [31].

**D1099Y** is located at possible interaction site and exchanges the medium size and acidic residue to the large and aromatic tyrosine. Surface residues not at defined interfaces are usually preserved. Still, a moderate to highly conserved domain on the surface of the structure includes this nsSNP, which is associated to familial HDL deficiency [70, 75] and predicted as deleterious in our analysis.

The** W1699C**, located within the transmembrane domain, is accumulated within the cytoplasm and a small proportion reaches the plasma membrane [76]. It introduces a cysteine residue, which stimulates the formation of a new disulphide bridge able to disrupt the ABCA1 protein structure preventing its oligomerization and transference to the plasma membrane. Probably, W1699C retains some residual functions, as shown by the plasma HDL-c levels found in members carrying this mutation which were not as low as might be expected in carriers [76]. In silico analysis with PolyPhen2 and MutPred indicate a deleterious effect of this nsSNP on ABCA1 function.

#### 3.2.2. Controversial Results for Prediction of the Neutral nsSNPs in the ABCA1 Gene

The mutant** R1897W** that induces a change from basic (R) to aromatic (W) is predicted functionally neutral in this analysis. This variant was identified in the mother and the brother of an FHA patient, who had plasma HDL levels in the lower range of the normal values [77].

Both the** D1289N** and** P2150L** variants identified in TD patients are considered as disease causative [42, 78, 79]. Further experimental evidences disagreeing with these results suggest that both could be nonfunctional variants [51, 54, 80]. Indeed, they showed a lipid transport activity, apoA1 binding, and distribution similar to WT [54, 80]. Interestingly, P2150L is only found in patients who also harbor a second variant, the deleterious** R587W** described above [54]. Besides, TD patients with D1289N variant were homozygous for a second mutation R2081W that could cause the shown pathological phenotype [79].** R2081W** is missed at the plasma membrane and instead accumulated intracellularly [54]. Our results suggest that mutations R2081W and R587W are highly deleterious. For D1289N and P2150L variants, PolyPhen2 predicts a neutral impact on protein function contrary to MutPred predictions that indicates a high probability for these mutants to be deleterious. The positions 1289 and 2150 are conserved among all ABCA1 orthologs but with the close-related ABCA7 and ABCA4. Since conservation patterns in ABCA1 protein endure for a relatively short time in evolutionary path, it is hard to determine if the conservation at these positions is due to functional constraint or simply reflects random chance. Along with experimental data, this suggests that R2081W is a major responsible of ABCA1 protein dysfunction found in TD patients.

The rare** R219K** polymorphism is located on an N-terminal extracellular loop, which mediates ABCA1 protein interaction with apoA1 [39, 56, 58, 59]. Despite high number of case-control studies conducted to investigate the functionality of R219K variant the results have been inconclusive [60, 81–83]. While some reports suggest an association of R219K is with risk of CVD [84], other research indicates a decreased atherosclerosis progression in general population [60, 85]. Conversely, large prospective studies found no association with HDL-c levels or atherosclerosis susceptibility [82, 86]. A meta-analysis indicates that R219K polymorphism is protective against CVD in Asians but not in Caucasians [87]. Unexpectedly, the K219 allele was associated with a decreased risk of myocardial infarction [18, 60, 84]. Also, this variant has effect on triglycerides [60] but not with HDL-c [84] or with apoA1 levels [85]. Otherwise, a study indicates that blood lipid levels do not seem to be R219K dependent [88]. Whether this variant confers major susceptibility to CVD is for clarification. The association of R219K variant to risk of AD has been studied in diverse ethnic groups [18–20, 89, 90]. Although conflicting results were noted, a study observed a protective dependence in delaying the risk of late-onset AD [18]. Equal to other cases, experimental results inconclusive and contradictories result prediction of the R219K polymorphism was predicted to be neutral in our in silico analysis.

Some studies [60, 91–93] but not all [39, 94] indicate that** I883M** variant severely increases the risk of atherosclerosis and AD [20]. The I883M has been reported as a milder phenotype with a significant reduction of HDL-c and cholesterol efflux (~70% of WT) [51]. In contrast, others studies [28, 60, 82, 83, 88] did not find any difference in lipid levels in I883M carriers. Studies among different healthy people [95, 96] as well as population with T2D [97] correlated the I883M variant with higher HDL-c concentration. Also, a stepwise regression approach identified I883M as one the key predictors of ischemic heart disease, whereas additive effects were found for V771M/I883M and I883M/E1172D pairs [82, 98]. As well, several studies have reported associations between V825I/I883M and increased plasma HDL-c levels [39, 67, 99]. Despite the controversial experimental results on the influence on cholesterol efflux activity observed of this polymorphism, our data predict that the I883M variant is functionally neutral. Interestingly, both alleles are found in the human population and the minor allele, methionine, is likely to be the ancestral allele at this position. Along with the human ABCA1orthologs, murine aligns valine at this position and the chimpanzee sequence aligns methionine. This divergence could explain why a simple conservation-based approach predicts the I883M change as neutral.

The** R1851Q** variant exchanges the large size and basic arginine (R) residue to medium size and polar glutamine predicted deleterious by MutPred and neutral by PolyPhen2. R1851Q occurs within the extracellular loop proximal to the transmembrane [68, 100]. Heterozygotes states show low HDL-c and apoA1 levels compared with those related to WT protein.

The** R230C** variant, found in Native American groups but not in European, Asian, or African individuals, has been associated with low levels of HDL-c and apoA1 [101]. These results are confirmed after adjusting for gender, BMI, and waist circumference [102]. Besides, the C230 allele is associated with obesity, metabolic syndrome, and T2D in Mexican population [101]. Still, R230C may have conferred resistance against certain infectious diseases [101]. R230C has been reported as a rare variant causing FHA in an Oji-Cree individual [67]. MutPred predicts a high probability of functional impairment of R230C, while that the PolyPhen2 program predicts the variant as neutral. Other facts that suggest functionally damage are (1) R230C occurs at the first extracellular loop, where TD and FHA mutations are clustered; (2) the arginine at position 230 is conserved between species; and (3) very different nature of residues involved; whereas arginine is basic and hydrophilic, the hydrophobic cysteine is vulnerable to disulfide bond.

The variants,** R1901S** that induces a change from large size and basic (R) to small size and polar (S);** Q2196H** that exchanges residues with similar physicochemical property (medium size, polar); and** E284K** that exchanges a medium size and acidic (E) to large size and basic (K), are predicted to be deleterious by MutPred and neutral by PolyPhen2. The R1901S and Q2196H variants occur within the C-terminal domain, close to the NBD, and E284K was located in the first extracellular loop, all of them associated to FHA [76]. The** A594T**,** I659V**,** T1512M**, and** R2004K** polymorphisms display different degrees of mislocalization to the plasma membrane and slight impacts on cholesterol efflux [103]. These nsSNPs were identified in low-HDL subjects [29]. The A594T, I659V, and T1512M were predicted to be functionally neutral and the R2004K mutation possibly damaging [29]. Finally, the novel mutation (**P85L**) in ABCA1 was identified in one family with low HDL but was not detected in over 400 chromosomes of healthy subjects [104]. Our in silico prediction indicated this variant as possible damaging by MutPred and neutral by PolyPhen2.

## 4. Conclusion and Future Directions

The practice of medicine, including health promotion and disease prevention, is primarily based on phenotype-based approaches. Most of them are proximal phenotypes achieved through biochemical markers. Finding genetic determinants of the phenotypes could not only clarify biological and functional consequence of variants but also might translate and extend to clinical phenotype. This focus would consider the large locus heterogeneity and numerous nongenetic factors to contribute to the phenotype. Since high levels of HDL-c are atheroprotective, there is extensive interest in developing agents that enhance ABCA1 expression and thereby raise plasma HDL-c levels. Amino acid exchange variants are crucial indicators of action sites and effective potential therapeutic approaches. In fact, nsSNPs represent disease modifiers capable of altering drug/nutrient response and potential targets vulnerable to environmental factors.

Evaluation of 233 nsSNPs (rare or common) found in ABCA1 transporter indicates that the rare 29 (12.44%) of them resulted to be highly deleterious with a probability >0.8. From 20 sequence variants found in about 16% of individuals with low HDL cholesterol only nine of them, D1706N, R1615P, W590L, C1477R, N1800H, R638Q, T2073A, A1670T, and S1731C, were predicted by MutPred and PolyPhen2 as functionally impaired. We have observed that mostly nsSNPs with verified functional effect in all experimental studies made are correctly predicted as damage variants by MutPred and PolyPhen2 tools. However, controversial experimental data are obtained for those nsSNPs predicted as neutral by both methods. Presumably clinical phenotype is the result of the additive effects and interactions among multiple alleles with different effect degree. Multiple rare alleles in ABCA1 contribute to plasma HDL-c levels in the general population.

Predicting the phenotypic consequence of nsSNPs using computational algorithms provides a better understanding of genetic differences in susceptibility to diseases and drug/nutrient response. These methods predict whether an amino acid altering mutation is deleterious or disease-causing based on physicochemical properties, population frequency, protein structure, and cross-species conservation. However, computational prediction tools are generally based on machine learning algorithms, which need to be trained before classifying a mutation as either neutral or deleterious. A major obstacle of these approaches is the lack of experimentally validated and impartial data sets. A further complication is that mutations in highly conserved sequences do not always produce phenotypes that are easily noticeable. Besides, knowledge of protein structure is crucial to accurately predict functional nsSNPs and understand their linkage with disease. Severe limitation arises thus when protein 3D-structure is not available as the ABCA1 case. Thus, an accurate, efficient, and generally applicable approach is needed to establish a genotype/phenotype correlation. Whole genome sequencing is likely to become a commodity that could be readily available at a reasonable cost and be easily accommodated into the decision making tree of health care of every individual. The challenging task will be to identify variants that are disease-causing or likely disease-causing and develop strategies to prevent and attenuate the evolving phenotype. Likewise, various complementary studies, genetic and biological, would be necessary to discern the associated alleles from the true disease-causing variants. Moreover a better understanding of genome components, such as functional, large intergenic noncoding RNAs, small noncoding RNAs, and primary transcripts, would be essential. An integrated approach that utilizes genomics, transcriptomic, proteomics, and metabolomic would be expected to facilitate identification and characterization of the mechanisms involved in the pathogenesis of the phenotype.

## Figures and Tables

**Table 1:** Structural and functional properties used by MutPred and PolyPhen2.

	PolyPhen2	MutPred
Sequence based properties		
Bond annotation	X	
Functional site annotation	X	X
Region annotation	X	X
PHAT score	X	
PSIC score	X	
SIFT score		X
Evolutionary attributes	X	X
Structural properties		
Secondary structure	X	X
Solvent-accessible surface area	X	X
Phi-psi dihedral angles	X	
Normalized accessible surface area	X	
Change in accessible surface propensity	X	
Change in residue side chain volume	X	
Region of the phi-psi map (Ramachandran map)	X	
Normalized B-factor	X	X
Ligand contacts	X	
Interchain contacts	X	
Functional site contacts	X	
Molecular Recognition Fragments (MoRFs)		X

**Table 2:** Functional effects of nsSNPs for ABCA1 gene (UniProt: O95477) predicted by PolyPhen2 and MutPred. A score >0.5 means that the mutation is classified as damaging by the algorithms. In addition, MutPred algorithm formulates hypotheses about structural and functional impact of the mutation; the most statistically significant hypothesis is reported in this table. The MAP column includes Minor Allele Frequency. The deviation column represents how close the two predicted scores are; a value of 0 corresponding to identical values.

Mutation	pph2_prob	Polyphen class	RFscore	Mutpred hypothesis	MAF	Deviation
A1010V	0,104	neutral	0,816	Loss of ubiquitination at K1009	NA	0,50346003
A1046D	0,999	deleterious	0,938	Loss of MoRF binding	NA	0,04313351
A1182T	0,001	neutral	0,425	Loss of sheet	0,00045914	0,29981328
A1407T	0,119	neutral	0,277	Gain of helix	0,00045914	0,11172287
A1631V	0,401	neutral	0,321	Loss of sheet	NA	0,05656854
A1670T	0,997	deleterious	0,566	Gain of glycosylation at A1670	NA	0,30476302
A1756T	0,81	deleterious	0,418	Gain of glycosylation at A1756	NA	0,27718586
A2028V	0,146	neutral	0,564	Gain of methylation at K2023	0	0,29557064
A255T	0	neutral	0,755	Loss of helix	NA	0,53386562
A343V	0,001	neutral	0,227	Loss of disorder	NA	0,15980613
A391S	0,318	neutral	0,34	Gain of disorder	NA	0,01555635
A594T	0,02	neutral	0,406	Loss of catalytic residue at A594	NA	0,27294322
A697T	0,416	neutral	0,508	Gain of sheet	0,00045914	0,06505382
A746G	1	deleterious	0,608	Loss of stability	0,00045914	0,27718586
A779T	0,411	neutral	0,468	Loss of helix	NA	0,04030509
A795S	0,07	neutral	0,494	Loss of helix	NA	0,29981328
A937V	0,991	deleterious	0,921	Loss of disorder	NA	0,04949748
C1477F	1	deleterious	0,496	Loss of catalytic residue at C1477	NA	0,35638182
C1477R	1	deleterious	0,852	Gain of methylation at C1477	NA	0,10465181
C1660R	0,954	deleterious	0,917	Gain of methylation at C1660	NA	0,02616295
C3S	0,005	neutral	0,48	Gain of disorder	NA	0,33587572
C887F	0,002	neutral	0,604	Gain of phosphorylation at S884	0,00137741	0,42567828
D1018G	0,992	deleterious	0,535	Loss of catalytic residue at D1018	NA	0,3231478
D1099N	1	deleterious	0,906	Gain of sheet	NA	0,06646804
D1099Y	1	deleterious	0,946	Loss of disorder	NA	0,03818377
D1263E	0,003	neutral	0,214	Gain of helix	NA	0,14919953
D1289N	0,339	neutral	0,573	Gain of sheet	NA	0,16546299
D1553E	0,001	neutral	0,273	Loss of helix	NA	0,19233304
D1706N	1	deleterious	0,611	Loss of helix	NA	0,27506454
D2243E	0,078	neutral	0,491	Gain of relative solvent accessibility	0,00459137	0,2920351
D434E	0,005	neutral	0,413	Gain of disorder	NA	0,28849957
D441N	0	neutral	0,348	Loss of helix	NA	0,24607316
D444E	0	neutral	0,338	Loss of helix	NA	0,23900209
D446E	0	neutral	0,301	Loss of helix	NA	0,21283914
D457E	0,001	neutral	0,566	Loss of helix	NA	0,39951533
D462G	0,071	neutral	0,555	Loss of catalytic residue at D462	NA	0,34223968
D581E	0,38	neutral	0,346	Loss of helix	NA	0,02404163
D677E	0,158	neutral	0,846	Loss of loop	NA	0,48648947
D831N	0,999	deleterious	0,589	Gain of helix	NA	0,28991378
D917N	0,1	neutral	0,577	Gain of MoRF binding	NA	0,33728994
E1005K	0,026	neutral	0,542	Gain of MoRF binding	NA	0,3648671
E1172D	0,003	neutral	0,253	Loss of sheet	0,0509642	0,1767767
E1172G	0,003	neutral	0,281	Loss of sheet	0,00091827	0,19657569
E1253K	0,048	neutral	0,453	Gain of ubiquitination at E1253	NA	0,28637825
E1916A	0,027	neutral	0,34	Gain of MoRF binding	0,00183655	0,22132442
E226G	0,019	neutral	0,47	Loss of stability	NA	0,31890516
E284K	0,206	neutral	0,842	Gain of methylation at E284	NA	0,44971991
E815G	0,072	neutral	0,361	Loss of disorder	NA	0,20435386
E868K	0,051	neutral	0,69	Gain of methylation at E868	0,00275482	0,45184123
F1285L	0,001	neutral	0,235	Loss of loop	NA	0,16546299
F1573S	0,065	neutral	0,532	Gain of disorder	NA	0,33021887
F2009S	0,811	deleterious	0,932	Gain of disorder	NA	0,08555992
F2082V	0,266	neutral	0,442	Gain of MoRF binding	NA	0,12445079
F2163S	0,939	deleterious	0,727	Loss of sheet	NA	0,14990664
F346L	0	neutral	0,239	Gain of disorder	NA	0,16899852
F426L	0,94	deleterious	0,549	Gain of helix	0,00045914	0,27647875
F632C	0,998	deleterious	0,694	Loss of stability	0,00045914	0,21496046
F950S	0,995	deleterious	0,664	Gain of disorder	NA	0,23405235
G1049R	1	deleterious	0,638	Gain of MoRF binding	NA	0,25597266
G1216V	0,951	deleterious	0,844	Loss of ubiquitination at K1214	NA	0,07566043
G1321A	0,936	deleterious	0,715	Gain of catalytic residue at G1321	NA	0,15627061
G156V	0,017	neutral	0,389	Loss of helix	NA	0,26304372
G2147E	0,49	neutral	0,397	Loss of catalytic residue at S2148	NA	0,06576093
G315W	0,997	deleterious	0,526	Loss of disorder	NA	0,33304729
G616V	0,998	deleterious	0,444	Loss of disorder	NA	0,39173716
G788W	1	deleterious	0,664	Loss of catalytic residue at P784	NA	0,23758788
G851R	0,998	deleterious	0,71	Gain of catalytic residue at G851	NA	0,20364675
H263D	0	neutral	0,399	Loss of helix	NA	0,28213561
H551D	0,613	deleterious	0,468	Gain of sheet	NA	0,10253048
I1084V	0,241	neutral	0,511	Gain of MoRF binding	NA	0,19091883
I1239V	0,078	neutral	0,376	Loss of catalytic residue at L1244	NA	0,21071782
I1517R	0,962	deleterious	0,917	Gain of catalytic residue at I1517	NA	0,03181981
I1555T	0,439	neutral	0,484	Loss of stability	NA	0,03181981
I1911T	0,006	neutral	0,437	Loss of stability	0,00045914	0,30476302
I35V	0,066	neutral	0,31	Loss of helix	0,00045914	0,17253406
I546V	0	neutral	0,31	Gain of MoRF binding	NA	0,2192031
I560T	0,829	deleterious	0,496	Loss of stability	NA	0,23546656
I649F	0,97	deleterious	0,669	Loss of catalytic residue at I649	NA	0,21283914
I659V	0,034	neutral	0,405	Loss of methylation at K663	NA	0,26233662
I883M	0,002	neutral	0,296	Gain of disorder	0,365473	0,20788939
K1250R	0,118	neutral	0,377	Loss of methylation at K1250	0,00045914	0,18314066
K1587R	0,034	neutral	0,377	Gain of helix	0,410927	0,24253763
K166R	0	neutral	0,251	Loss of ubiquitination at K166	NA	0,1774838
K1761T	0,038	neutral	0,545	Loss of methylation at K1761	0,00045914	0,35850314
K1974R	0,158	neutral	0,679	Loss of ubiquitination at K1974	NA	0,36840263
K2040E	0,243	neutral	0,459	Loss of MoRF binding	0,00045914	0,15273507
K401Q	0,007	neutral	0,322	Loss of ubiquitination at K401	NA	0,22273864
K613E	0,002	neutral	0,428	Loss of ubiquitination at K613	0,00045914	0,30122749
K776N	0,985	deleterious	0,568	Loss of methylation at K776	0,00183655	0,29486353
L1041V	0,999	deleterious	0,625	Loss of ubiquitination at K1040	0,00137741	0,26445794
L1379F	0,987	deleterious	0,866	Loss of catalytic residue at L1379	NA	0,08555992
L1408F	0,443	neutral	0,418	Loss of stability	0,00045914	0,01767767
L1648P	0,961	deleterious	0,749	Loss of stability	NA	0,14990664
L184S	0,007	neutral	0,576	Loss of sheet	0,00045914	0,40234376
L184W	0,928	deleterious	0,54	Gain of helix	0,00045914	0,27435743
L2032M	0,897	deleterious	0,688	Gain of methylation at K2036	NA	0,14778532
L2168P	0,984	deleterious	0,693	Gain of disorder	NA	0,20576807
L2187Q	0,995	deleterious	0,698	Gain of disorder	0,00045914	0,21001071
M1012I	0,008	neutral	0,595	Loss of catalytic residue at V1008	NA	0,41507168
M1091T	0,98	deleterious	0,955	Gain of disorder	NA	0,01767767
M233V	0	neutral	0,417	Loss of stability	NA	0,29486353
M415L	0,002	neutral	0,423	Gain of relative solvent accessibility	NA	0,29769196
M674L	0,534	deleterious	0,599	Loss of catalytic residue at M674	0,00045914	0,04596194
N1185K	0,01	neutral	0,583	Gain of MoRF binding	NA	0,40517219
N1185S	0,002	neutral	0,358	Gain of helix	NA	0,25173001
N1406K	0,006	neutral	0,408	Gain of ubiquitination at N1406	NA	0,28425693
N1611D	0,968	deleterious	0,921	Loss of MoRF binding	NA	0,03323402
N1800H	0,758	deleterious	0,826	Loss of stability	NA	0,04808326
N1800S	0,241	neutral	0,84	Loss of stability	NA	0,42355696
N2119Y	0,96	deleterious	0,727	Loss of MoRF binding	NA	0,16475588
N820S	0,001	neutral	0,348	Loss of catalytic residue at N820	NA	0,24536605
N935H	0,996	deleterious	0,966	Gain of disorder	NA	0,02121321
N935S	0,831	deleterious	0,969	Gain of disorder	NA	0,09758074
P1065S	0,998	deleterious	0,906	Gain of MoRF binding	NA	0,06505382
P1475S	1	deleterious	0,235	Gain of phosphorylation at P1475	NA	0,54093669
P1878T	0,019	neutral	0,482	Gain of helix	NA	0,32739044
P2150L	0,068	neutral	0,826	Loss of disorder	NA	0,53598694
P248A	0	neutral	0,207	Loss of helix	NA	0,1463711
P250L	0	neutral	0,244	Loss of glycosylation at P250	NA	0,17253406
P641L	1	deleterious	0,603	Gain of MoRF binding	NA	0,28072139
P855S	0,97	deleterious	0,59	Loss of catalytic residue at W856	NA	0,26870058
P85L	0,394	neutral	0,618	Loss of disorder	0,00045914	0,15839192
Q1279K	0,032	neutral	0,715	Gain of MoRF binding	NA	0,48295393
Q188K	0,001	neutral	0,543	Gain of ubiquitination at Q188	0	0,38325188
Q205E	0,01	neutral	0,255	Loss of loop	NA	0,17324116
Q205R	0	neutral	0,279	Gain of helix	NA	0,19728279
Q2196H	0,011	neutral	0,798	Loss of MoRF binding	NA	0,55649304
Q2210H	0,984	deleterious	0,388	Gain of catalytic residue at D2214	NA	0,42143564
Q597R	1	deleterious	0,71	Gain of MoRF binding	NA	0,20506097
Q849R	0,689	deleterious	0,662	Gain of methylation at Q849	NA	0,01909188
R104C	0,985	deleterious	0,589	Loss of MoRF binding	NA	0,28001429
R1068C	1	deleterious	0,962	Loss of MoRF binding	NA	0,02687006
R1068H	1	deleterious	0,972	Loss of MoRF binding	NA	0,01979899
R1082C	1	deleterious	0,694	Loss of MoRF binding	NA	0,21637468
R1195Q	0,044	neutral	0,364	Loss of loop	NA	0,22627417
R1195W	0,952	deleterious	0,589	Gain of catalytic residue at A1194	NA	0,25667976
R126H	0	neutral	0,467	Loss of MoRF binding	NA	0,33021887
R1273L	0,007	neutral	0,35	Loss of loop	NA	0,24253763
R1283C	0,001	neutral	0,449	Gain of ubiquitination at K1278	NA	0,31678384
R1341T	0,016	neutral	0,604	Loss of methylation at R1341	≤0,001	0,41577879
R1344W	0,993	deleterious	0,64	Loss of methylation at K1345	0,00045914	0,24960869
R1417H	0,837	deleterious	0,457	Loss of phosphorylation at T1416	0,00183655	0,26870058
R1615P	0,947	deleterious	0,895	Loss of methylation at R1615	≤0,001	0,03676955
R1615Q	0,187	neutral	0,679	Gain of helix	NA	0,34789654
R1680Q	0,997	deleterious	0,807	Gain of ubiquitination at K1683	NA	0,13435029
R1680W	1	deleterious	0,911	Loss of disorder	NA	0,06293251
R1839H	0,006	neutral	0,48	Loss of phosphorylation at S1842	0,00045914	0,33516861
R1851Q	0,015	neutral	0,897	Gain of catalytic residue at R1851	NA	0,62366818
R1897W	0,009	neutral	0,408	Probably damaging	NA	0,28213561
R1901S	0,091	neutral	0,81	Gain of phosphorylation at R1901	NA	0,50840978
R1925Q	0,006	neutral	0,69	Loss of MoRF binding	0,00229568	0,48366104
R2004K	0,911	deleterious	0,699	Gain of ubiquitination at R2004	NA	0,14990664
R2030Q	0,032	neutral	0,442	Loss of MoRF binding	NA	0,28991378
R2081W	0,998	deleterious	0,936	Loss of MoRF binding	NA	0,04384062
R2173Q	0,008	neutral	0,382	Gain of sheet	NA	0,26445794
R2189G	0,142	neutral	0,443	Loss of MoRF binding	NA	0,21283914
R219K	0	neutral	0,41	Gain of ubiquitination at R219	0,419192	0,28991378
R230C	0	neutral	0,68	Loss of MoRF binding	0,00734619	0,48083261
R306G	0,04	neutral	0,463	Loss of helix	≤0,001	0,29910617
R306H	0,899	deleterious	0,431	Loss of helix	NA	0,33092597
R369H	0,937	deleterious	0,433	Loss of MoRF binding	NA	0,35638182
R437Q	0,088	neutral	0,48	Loss of helix	NA	0,27718586
R437W	0,991	deleterious	0,562	Gain of catalytic residue at L435	NA	0,30334881
R443K	0	neutral	0,44	Loss of stability	0,00045914	0,31112698
R496W	0,209	neutral	0,576	Loss of loop	NA	0,25950819
R500H	0,013	neutral	0,519	Loss of helix	NA	0,35779603
R587W	1	deleterious	0,688	Loss of disorder	NA	0,22061732
R638Q	0,957	deleterious	0,664	Loss of methylation at R638	NA	0,20718229
R638W	0,999	deleterious	0,671	Loss of methylation at R638	0,00045914	0,23193102
R666Q	1	deleterious	0,437	Gain of ubiquitination at K668	NA	0,39810112
R666W	1	deleterious	0,56	Gain of catalytic residue at R666	NA	0,31112698
R672Q	0,832	deleterious	0,501	Loss of MoRF binding	NA	0,23405235
R965C	0,302	neutral	0,608	Loss of disorder	≤0,001	0,21637468
R999C	0,127	neutral	0,766	Loss of solvent accessibility	NA	0,45184123
R999L	0,208	neutral	0,748	Loss of solvent accessibility	NA	0,38183766
S107A	0,002	neutral	0,342	Gain of helix	NA	0,24041631
S1141Y	0,805	deleterious	0,306	Loss of phosphorylation at S1141	NA	0,35284628
S1157N	0,022	neutral	0,355	Gain of sheet	0,00045914	0,23546656
S116N	0,196	neutral	0,241	Loss of phosphorylation at S116	NA	0,03181981
S1181F	0,042	neutral	0,542	Loss of disorder	0,00045914	0,35355339
S1255R	0,027	neutral	0,326	Loss of glicosilation at S1255	0,00183655	0,21142493
S1280R	0,009	neutral	0,351	Gain of sheet	0,00045914	0,24183052
S1376G	0,007	neutral	0,407	Gain of methylation at K1373	≤0,001	0,28284271
S139G	0	neutral	0,284	Gain of helix	NA	0,20081833
S1506L	0,996	deleterious	0,733	Loss of helix	NA	0,18596908
S1536F	0,998	deleterious	0,594	Loss of disorder	NA	0,28567114
S1731C	0,893	deleterious	0,526	Loss of helix	≤0,001	0,25950819
S212T	0,028	neutral	0,375	Gain of helix	0,00459137	0,24536605
S2182F	0,578	deleterious	0,498	Loss of disorder	NA	0,05656854
S2186F	0,862	deleterious	0,458	Loss of disorder	NA	0,28567114
S442R	0,001	neutral	0,449	Loss of helix	NA	0,31678384
S713G	1	deleterious	0,576	Loss of stability	NA	0,29981328
S780N	0,996	deleterious	0,559	Loss of stability	NA	0,30900566
T1175M	0,432	neutral	0,331	Loss of sheet	0,00091827	0,07141779
T1242M	0,993	deleterious	0,62	Loss of catalytic residue at T1242	NA	0,26375083
T1399M	0,003	neutral	0,279	Loss of glycosylation at T1399	NA	0,19516147
T1401I	0,003	neutral	0,368	Loss of glycosylation at T1401	NA	0,25809398
T1427M	0,005	neutral	0,352	Gain of catalytic residue at T1427	0,00091827	0,24536605
T2073A	0,852	deleterious	0,657	Loss of phosphorylation at T2073	≤0,001	0,13788582
T459P	0,03	neutral	0,534	Gain of helix	≤0,001	0,35638182
T515A	0,04	neutral	0,449	Gain of helix	NA	0,28920667
T774P	0,003	neutral	0,61	Gain of methylation at K776	0,00183655	0,42921382
T929I	0,946	deleterious	0,889	Loss of disorder	NA	0,04030509
T940M	1	deleterious	0,844	Loss of methylation at K939	NA	0,11030866
V1054I	0,951	deleterious	0,51	Loss of ubiquitination at K1052	NA	0,31183409
V1096I	0	neutral	0,467	Gain of helix	NA	0,33021887
V1158I	0,065	neutral	0,226	Loss of sheet	NA	0,11384419
V1674I	0,053	neutral	0,376	Loss of catalytic residue at V1674	0,00137741	0,22839549
V1704D	0,752	deleterious	0,876	Loss of helix	NA	0,08768124
V1806M	0,98	deleterious	0,417	Gain of ubiquitination at K1804	NA	0,39810112
V1858M	0,305	neutral	0,472	Loss of catalytic residue at V1858	NA	0,11808683
V2035M	0,042	neutral	0,38	Loss of methylation at K2036	NA	0,23900209
V2244I	0,002	neutral	0,386	Gain of sheet	NA	0,271529
V304M	0,823	deleterious	0,387	Gain of disorder	NA	0,30829856
V380I	0,001	neutral	0,353	Gain of methylation at K376	NA	0,24890159
V399A	0,071	neutral	0,563	Gain of disorder	0,00183655	0,34789654
V408G	0,101	neutral	0,431	Loss of stability	NA	0,23334524
V481L	0,001	neutral	0,374	Gain of sheet	0,00045914	0,26375083
V589I	0,004	neutral	0,334	Gain of catalytic residue at V589	0,00091827	0,23334524
V654G	0,433	neutral	0,438	Loss of stability	NA	0,00353553
V702A	0,002	neutral	0,408	Loss of stability	NA	0,28708535
V724M	0,833	deleterious	0,462	Loss of catalytic residue at V724	0,00045914	0,26233662
V771M	0,034	neutral	0,421	Loss of ubiquitination at K776	0,0606061	0,27365032
V825I	0,001	neutral	0,401	Loss of catalytic residue at V825	0,128099	0,28284271
W1699C	1	deleterious	0,915	Gain of catalytic residue at L1700	NA	0,06010408
W590L	0,841	deleterious	0,888	Loss of helix	≤0,001	0,03323402
W590S	0,889	deleterious	0,857	Gain of disorder	≤0,001	0,02262742
W840R	0,998	deleterious	0,923	Gain of methylation at W840	NA	0,05303301
Y1921H	0,986	deleterious	0,752	Gain of disorder	NA	0,16546299
Y2178H	0,982	deleterious	0,826	Gain of disorder	NA	0,11030866
Y2206D	0,992	deleterious	0,878	Gain of sheet	NA	0,08061017
Y482C	0,03	neutral	0,826	Gain of loop	NA	0,56285691
Y835H	0,967	deleterious	0,653	Loss of stability	NA	0,22203153
